# Assessing the Impact of Mechanical Damage on Full-Thickness Porcine and Human Skin Using an *In Vitro* Approach

**DOI:** 10.1155/2015/434623

**Published:** 2015-07-13

**Authors:** Hinda Dabboue, Nicolas Builles, Éric Frouin, Dan Scott, Jeanne Ramos, Gilberte Marti-Mestres

**Affiliations:** ^1^Faculty of Pharmacy, IBMM-UMR 5247, University of Montpellier, France; ^2^Tissue Bank and CCBHM, Saint Eloi Hospital, Montpellier, France; ^3^Pathology Department, University Hospital of Poitiers, France; ^4^Service of Anatomy and Cytopathology, Gui de Chauliac Hospital, CHU, University of Montpellier, France

## Abstract

For most xenobiotics, the rates of percutaneous absorption are limited by diffusion through the horny layer of skin. However, percutaneous absorption of chemicals may seriously increase when the skin is damaged. The aim of this work was to develop an *in vitro* representative model of mechanically damaged skins. The epidermal barrier was examined following exposure to a razor, a rotating brush, and a microneedle system in comparison to tape-stripping which acted as a reference. Excised full-thickness skins were mounted on a diffusion chamber in order to evaluate the effect of injuries and to mimic physiological conditions. The transepidermal water loss (TEWL) was greatly increased when the barrier function was compromised. Measurements were made for all the damaged biopsies and observed histologically by microscopy. On human and porcine skins, the tape-stripping application (0 to 40 times) showed a proportional increase in TEWL which highlights the destruction of the stratum corneum. Similar results were obtained for all cosmetic instruments. This is reflected in our study by the nonsignificant difference of the mean TEWL scores between 30 strips and mechanical damage. For a specific appreciation, damaged skins were then selected to qualitatively evaluate the absorption of a chlorogenic acid solution using fluorescence microscopy.

## 1. Introduction

The primary property of the skin is to act as a barrier function. The outermost epidermal layer, the stratum corneum (SC), is an effective barrier that protects against external aggression and prevents the delivery of xenobiotic molecules across the skin [1–5].

Due to skin barrier properties, a chemical must exhibit specific physicochemical traits, that is, a low molecular weight, a low melting point, and a logP (octanol-water partition coefficient) from 1 to 4, in order to be a candidate for passive transepidermal delivery [6–8]. To overcome significant barrier properties of the stratum corneum, numerous approaches were conducted in the pharmaceutical domain to enhance percutaneous penetration of drugs, such as nanoformulations [9, 10] and by production of temporary [11] or permanent holes [12] in the skin. The use of these techniques has now advanced to the field of cosmetics. A large number of instruments, apparatus, and devices are now marketed as “high-tech beauty gadgets” that are claimed to smooth wrinkles as well as renew and temporarily alter the appearance of the face and skin [13]. Cosmetics and cosmetic devices are used to improve appearance and should not impart any health benefits or permeate past the epidermal layer; otherwise they would be classified as a medicine. The skin as an outer organ is naturally susceptible to mechanical damage from its environment which can impair its barrier function, and this must be factored into the development and design of cosmetic gadgetry.

The aim of our study was to establish an* in vitro* model of acute barrier disruption, using Franz cell with full-thickness porcine and human skin [14–16], to investigate various types of skin damage, based on Fick's law of diffusion [17, 18].

Tape-stripping, first described by Fritsch et al. [19], is a robust method in SC physiology research. Adhesive films are pressed onto the surface of the skin with a fixed amount of pressure before removal [20]. The superficial layers of the SC adhere to the film, are stripped from the SC, and are then accessible for further investigation. At the same time, repeated tape-stripping may be an effective comparative model for impaired skin barrier function [21–23]. Transepidermal water loss (TEWL) was used as the unit of SC damage between models, measured in grams per centimeter squared per hour (g/cm^2^/h). The TEWL is widely used in skin integrity tests with a large historical dataset [24–28]. Many studies have suggested that high TEWL is associated with various skin diseases, including atopic dermatitis, psoriasis, contact dermatitis, and ichthyosis [29–32]. Thus, TEWL is thought to be a useful parameter that characterizes skin barrier function in man.* In vitro* experiments were performed using Franz cells with full-thickness porcine and human skin. Healthy pig ear skin was compared to healthy human skin with and without stretch marks because they are an excellent surrogate to human skin, due to physiological similarity and availability. The effects of selected 5 to 30 or 40 repeated tape-strippings were then compared to the other types of induced skin injury.

The impact of two new cosmetic “gadgets,” microneedles [33] and rotating brush [34], was studied in order to evaluate skin damage after their application. Influence of a conventional razor [35] was also investigated. Microneedles of 1 mm length disposed on a roller were studied with respect to the efficiency of skin perforation. Microneedles were initially used for skin disruption to facilitate transdermal drug delivery until recently. This device was then introduced in the cosmetic domain to treat scars, wrinkles, and stretch marks. The impact of a rotating brush used for face cleansing was also investigated* via* application to a fresh biopsy with a cosmetic gel containing salicylic acid. A manual razor was also applied three times on a biopsy in the same direction.

The purpose of the current work was to investigate the suitability of different skin integrity tests to differentiate impaired from intact human skin.

## 2. Materials and Methods

### 2.1. Skin Preparation

Porcine ears were obtained from freshly killed animals in a local slaughterhouse (Pézenas, France). After cleaning with cold tap water, full-thickness skin was removed with a scalpel from the cartilage of the outer region. Human skin was obtained from “Centre des Collections Biologiques Hospitalières, CHU (Central University Hospital), Montpellier” (biobank identification number BB-0033-00031) following official agreement compliant with French regulation and full written consent from donors. Human skin was retrieved from the plastic surgery unit (abdominoplasty), treated with povidone iodine antiseptic (PVP-I, Betadine) prior to extraction, harvested by a surgeon in a medical grade sterile pot system (Cryokit, Verreries Talanconnaises, France) with sterile NaCl 0.9% at +4°C (Sodium Chloride 0.9%, B/Braun, Melsungen, Germany), sent to the tissue bank where subcutaneous fat was removed up from the dermal layer, and conditioned within a Cryokit with NaCl 0.9% system. The skin was sent to the laboratory at +4°C up to 4 hours from retrieval, ensuring an optimal skin quality. All skins were inspected for visible skin lesions prior to use. Only intact healthy looking skin was used for experiments.

All skins were cut using a punch-biopsy in the laboratory (2 cm^2^ diameter) to fit Franz cells, and thickness was measured in each case using Mitutoyo 2050S apparatus (ranging between 1.0 and 1.4 mm) prior to labelling, freezing in aluminium foil, and storing at −20°C for a period not exceeding 4 months. Skin from different donors was used to demonstrate reproducibility of the study. A minimum of 4 different subjects was assigned to each group in order to minimize any individual variance which would interfere with overall outcome.

### 2.2. Types of Skin Damage

#### 2.2.1. Tape-Stripping

Standard sized D-Squame Skin Sampling Discs (22 mm^2^ diameter, Monaderm, Monaco) were applied to skin biopsies prior to application of 225 g/cm^2^ pressure during 3 seconds, provided by the D-Squame D500 apparatus applicator (Monaderm, Monaco). This process was subjected to affected skin samples, 5 to 40 times using fresh discs each time. The D-Squame tapes were peeled from different directions (90° each time) in rotation until the process was completed.

#### 2.2.2. Microneedles

A titanium Micro Needle Roller System (RoHS, CE) composed of 540 needles of 1 mm length was used. The instrument was rolled firmly onto the biopsies ten times vertically followed by ten times horizontally.

#### 2.2.3. Razor

Wilkinson Sword Extra Beauty 3 razors were used. They contained an aloe vera adjuvant alongside physiologic solution (NaCl 0.9%, Versylene, Fresinius Kabi, Sevres, France). The skin biopsies were shaved 3 times in the same direction without shaving formulation.

#### 2.2.4. Rotating Brush

Pureo Sonic Brush (Elle by Beurer, France) was applied to designated biopsies at the highest speed and rotated firmly around the skin for one minute at the highest speed. Integrity of the skin was further challenged following application of a cleansing gel. A pure active gel (20 *μ*L) containing salicylic acid and zinc gluconate was applied to previously moistened skin with saline and rubbed gently before being washed off 60 seconds after.

### 2.3. *In Vitro* Model

Glass Franz diffusion cells with average capacity of 9 mL ± 0.35 mL were used with a surface area of 1 cm^2^. Each cell was filled with saline solution (0.9% NaCl) representing the thermodynamic equivalent of fluid beneath the epidermis* in vivo*. Franz cells were thermostated at 37.2°C ± 1°C (Polystat CC1, Huber, Offenburg, Germany) with receptors stirred at 600 rpm/min with a magnetic bar throughout the experiment. Skin surface temperature was then measured at 32°C ± 1°C in order to confirm correlation to* in vivo* temperature prior to application of mechanical damage and before TEWL measurements. The complete model was held in place by clamps (Rotulex, Pyrex, SciLabware, Clichy, France).

### 2.4. TEWL Measurement

A Tewameter TM 300 was used (Monaderm, Monaco). A minimum of one hour was allowed for samples to equilibrate following direct application to Franz cell receptor temperature from a frozen state. After this time, the TEWL results were obtained (g·m^2^·h^−1^). In the case of deliberately damaged skin of deliberately damaged skin, TEWL measurements were taken on an intact biopsy and compared to the same biopsy 30 minutes after injuries were applied. Care was taken prior to measurement to ensure that there was an absence of air bubbles lying under the dermis in contact with the receptor fluid. TEWL measurements were conducted on average three times per sample from the top of the donor cell.

### 2.5. Histological Analysis

#### 2.5.1. Optical Microscopy

After mechanical damage, biopsies were fixed in 4% paraformaldehyde (Sakura Society, Tokyo, Japan) solution for up to 48 hours. Thereafter, skin samples were embedded in paraffin (Leica Society, Germany) and cross sections of 5 *μ*m were cut. After drying and deparaffining blades, cut sections were automatically colored by haematoxylin and eosin (Dako Society, Les Ulis, France). Slides (Superfrost Plus, VWR International, Fontenay-sous-Bois, France) that contained histological sections were placed on a motorized support under the Nanozoomer Slide Scanner (Hamamatsu). Then, the entire surface of the sample section was analyzed with NDP Nanozoomer software.

#### 2.5.2. Scanning Electron Microscopy

Skin biopsies were washed in PBS and fixed in a 2.5% glutaraldehyde (Electron Microscopy Sciences, Hatfield, PA, USA) and Sorensen phosphate buffer (0.133 M, Electron Microscopy Sciences, Hatfield, PA, USA) solution, pH 7.2, for an hour at room temperature and rinsed in Sorensen buffer. Samples were then dehydrated using a gradient ethanol series (30–100%), followed by critical point drying with CO_2_. Subsequently, samples were sputter-coated with an approximative 10 nm thick gold film and then examined under a scanning electron microscope (Hitachi S4000, at CRIC, Montpellier, France) using a lens detector with an acceleration voltage of 10 kV at calibrated magnifications.

#### 2.5.3. Fluorescence Microscopy

1% of chlorogenic acid (Sigma-Aldrich, St. Louis, USA) was solubilized in a mixture (2/8, v/v) of PEG-400 (Cooper, Melun, France) and methanol (Sigma-Aldrich, St. Louis, USA). 20 *μ*L of this solution were added to skin samples and incubated in a thermostated Franz cell for 24 hours. The donor compartment of the cell was covered with parafilm to prevent evaporation of the applied compounds. After 24 hours, skin samples were dried; surfaces were gently dabbed with methanol (Sigma Aldrich) using gauze prior to embedding in OTC matrix (CellPath, UK) and frozen in liquid nitrogen. The samples were then stored at −80°C until preparation of microscope slides using a cryostat. Six-micron cryo-cross-sections were observed by fluorescence microscopy (Leica DMR-Camera Leica DFC 310 FX, Nanterre, France) with a DAPI filter (excitation 350 nm and 450–490 nm emission) with few drops of Neu reagent [36] (1% of 2-aminoethyl-diphenylborinate) (Sigma-Aldrich, St. Louis, USA) in methanol (Sigma-Aldrich, St. Louis, USA), which potentiates the fluorescence of chlorogenic acid.

### 2.6. Statistical Analysis

Statistical calculations were performed by means of the PC program, Statgraphics-Centurion XVII [37]. A nonparametric Kruskal-Wallis (KW) was used with a box and whisker (BaW) representation. Notches are useful in offering a guide to significance of difference of medians, in the case that the notches of two boxes do not overlap. Bonferroni test was then used to show pairwise comparison between the average ranks of each group.

## 3. Results and Discussion

### 3.1. Comparison of Healthy Human and Pig Skins

**Figure 1:**
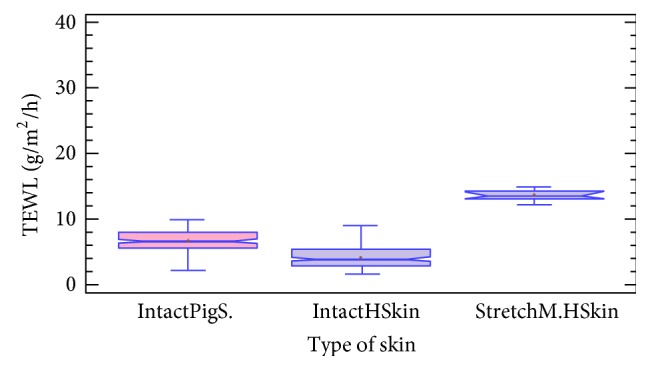
Box and whisker-plot, TEWL analysis, and comparison between healthy human (IntactHSkin) and porcine (IntactPigS.) skin and human stretch marks biopsies (StretchM.HSkin) (KW is equal to 105.55, *P* value = 0.00). +: mean.

As shown in Figure 1, intact pig and human skins were differentiated between biopsies with stretch marks. A KW nonparametric test was used, whereby this test does not require the assumption that all the samples were drawn from normally distributed populations with equal variance. The KW test for TEWL by type of skin is equal to 105.55 with a *P* value = 0.00, confirming that there was a statistically significant difference amongst the medians at a 95% confidence level. For the intact pig skin (*n* = 154), the average rank was 166.68 and for the intact human skin (*n* = 106) the average rank was 77.92. Finally, for the stretch mark human skin (*n* = 8), average rank was 264.5. All data were reported in Table 1. Graphically, the box and whisker procedure denoted a statistically significant difference between the 3 types of skin, which was corroborated by the Bonferroni procedure. We have demonstrated an important increase in the mean of TEWL for the stretch mark human skin (13.6 g/m^2^/h) compared to the two others, where the mean TEWL is 3 times greater than that of healthy human skin (4.2 g/m^2^/h) and almost twice as large as healthy pig skin (6.7 g/m^2^/h).

One hypothesis is that stretch marks are induced by excessive mechanical stretching of skin to the point of rupturing dermal elastic fibers and that local fibroblasts are unable to adequately repair or replace those extracellular matrix components that are solely responsible for the resilience of skin [38]. The presence of stretch marks on the human skin is equivalent to the presence of a lesion when comparing TEWL results. Therefore as human skin with stretch marks already corresponds to endogenous lesions that could increase TEWL, we decided not to use them in the following experiments. In contrast, excised porcine ear skin has been shown to be a suitable skin substitute model for human skin, based on morphological and functional data [39, 40]. However, sources for excised human skin for* in vitro* studies are limited.

### 3.2. Stripped Skin as Model

A control group with undamaged skin was compared with a group where epidermis of the biopsies was stripped 5, 10, 20, 30, and 40 times. The maximal number of adhesive tapes used was fixed to 40 for human and porcine skin. For comparison, two protocols were implemented in the study: in the first one (protocol 1) tape-stripping was applied 5 to 40 times successively on the same biopsy, while in the second one (protocol 2), a new skin biopsy was used each time.

Netzlaff et al. [41] have proven that TEWL measurement cannot detect small changes in the stratum corneum, but a clear increase in TEWL induced by the impairment of the SC barrier was expected.

**Figure 2:**
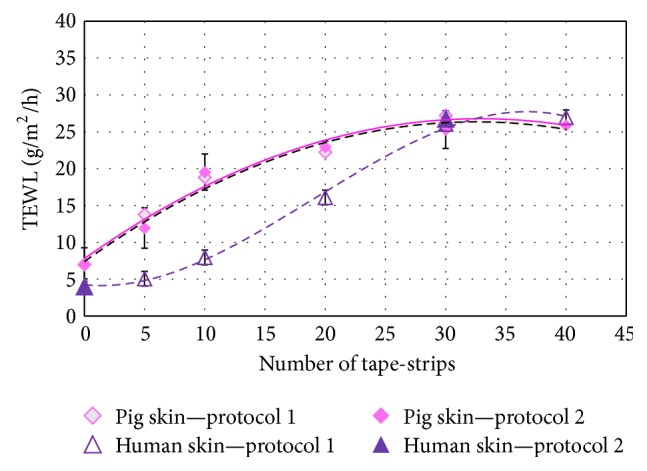
TEWL analysis and comparison between two protocols of tape-stripping and healthy porcine and human skin; protocol 1: tape-stripping followed in the same skin, protocol 2: tape-stripping on different biopsies.

With both protocols, there was a strong correlation between the number of stripping times and TEWL. And the removal of 30–40 tape-strips formed a plateau corresponding to removal of the last stratum corneum layers. At these steps, a 4-fold loss of barrier function for pig skin and a 6-fold loss of barrier function for human skin were observed.  Figure 2 illustrates the increase in TEWL as SC width decreases. The KW test for TEWL comparing protocol and skin is equal to 0.88 with a *P* value = 0.82, and there are no statistically significant differences amongst the medians at 95% confidence level (all data are reported in Table 2). This provides important information and all data obtained from protocols 1 and 2 were then pooled for neatest tests. This crucial step was corroborated by Rubio et al. who have shown that 20 and 35 strips cause, respectively, minor and major increases in TEWL and that more strips have nonsignificant effect [42].

**Figure 3:**
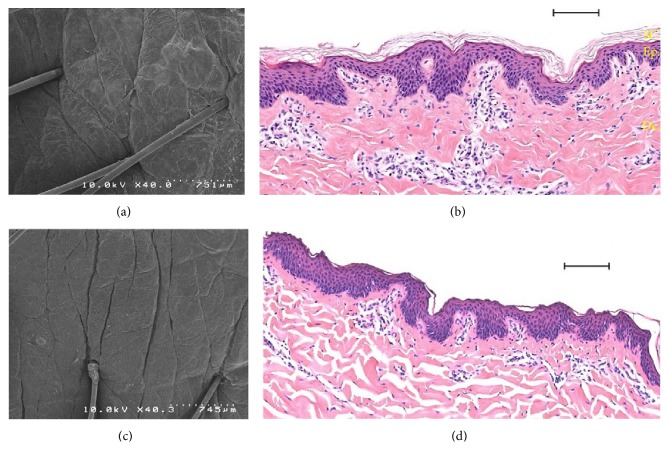
Histological analysis, comparison between porcine skin tape-stripped and no treatment by scanning electron microscopy (a, c), and optical microscopy of haematoxylin and eosin stained section (b, d). (a, b) Control porcine skin; (c, d) tape-stripping (×30) porcine skin. Scale bar (b, d): 100 *μ*m. SC: stratum corneum, Ep.: epidermis, and De.: dermis.

**Figure 4:**
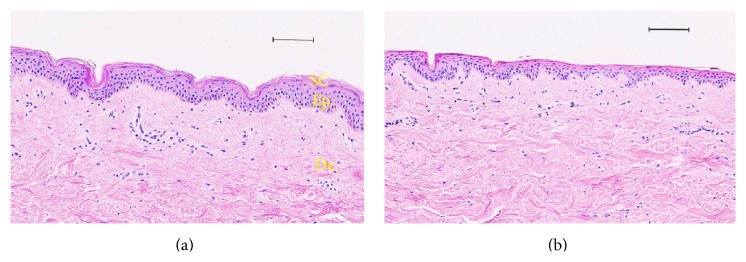
Histological analysis and comparison between a human skin tape-stripped and no treatment by optical microscopy (haematoxylin and eosin staining). (a) Human healthy skin; tape-stripping (×30) human skin (b). Scale bar: 100 *μ*m.

In order to visualize the destruction of the stratum corneum in porcine ear and human skins models, histological sections were investigated as shown in Figures 3 and 4, respectively. The stratum corneum is progressively removed by serial adhesive tape-stripping. With 40 strips drastic damage is observed on the epidermis. Thus 30 tape-strips were used as a realistic reference to damaged skin for further comparative studies.

Compared to intact skin, smoother skin surface was observed by scanning electron microscopy (SEM) (Figure 3(c)) in porcine samples. Such findings have explained the very small thickness of the stratum corneum after 30 tape-strips observed on optical microscopy images (Figure 3(d)). The same observations were made with human skin samples when 30-tape-strip damage was applied (Figure 4(b)). Thus we have herein developed a standardized model based on 30 tape-strips for evaluating skin injuries when the stratum corneum is impaired homogeneously. This method of tape-stripping application has often been used as a model of skin lesions in order to study the penetration of xenobiotics [43–48].

### 3.3. Mechanical Damage by Devices

With recent developments in the cosmetic industry with regard to device models, one trend is towards home use. But are these practices safe if cosmetic products are applied after their use? The Margin of Safety (MoS) of substances in a finished cosmetic product is derived by dividing the nonobserved adverse effect level (NOAEL) by the systemic exposure dosage (SED). Exposure scenario is based to an extent on the amount of substance that may be absorbed through the skin in order to calculate the SED [49]. Numerous complications may arise because SED is usually calculated with data of absorption obtained from chemical applications on healthy skin. In a risk assessment, the toxicity of the chemical is considered in conjunction with anticipated exposure levels for the target population. But with the use of cosmetics devices levels of SED are underestimated and will not represent the worst case compared to exposure.

**Figure 5:**
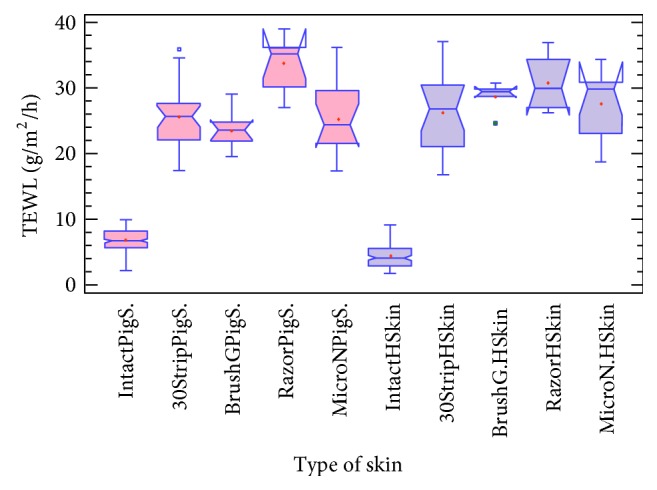
Box and whisker-plot, TEWL analysis with the different mechanical injuries, and comparison with the control biopsy in porcine and human skin (KW is equal to 256.59, *P* value = 0.00, +: mean, and □: outliers points).

**Figure 6:**
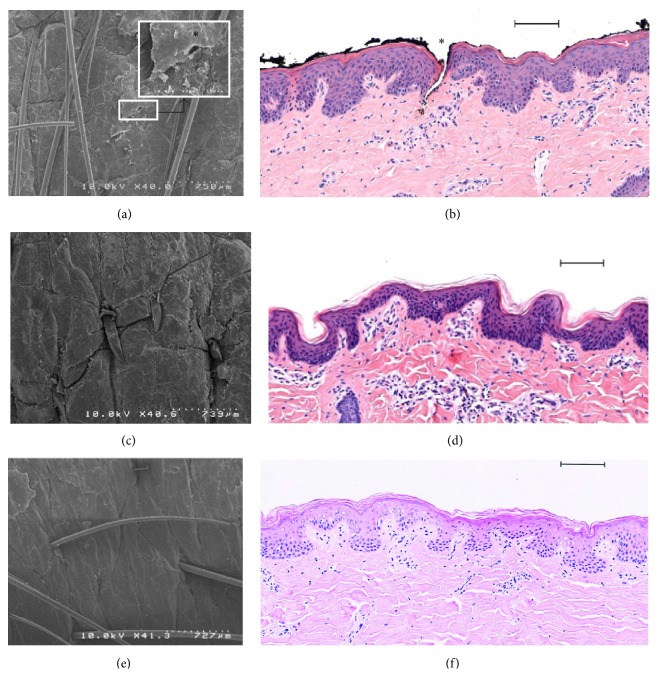
Histological analysis of damaged porcine skin. Scanning electron microscopy (a, c, and e) and optical microscopy haematoxylin and eosin staining (b, d, and f). (a, b) Microneedles application (^∗^microlancing); (c, d) razor and shaving; and (e, f) rotative brush application with the gel. Scale bar (b, d, and f): 100 *μ*m.

**Figure 7:**
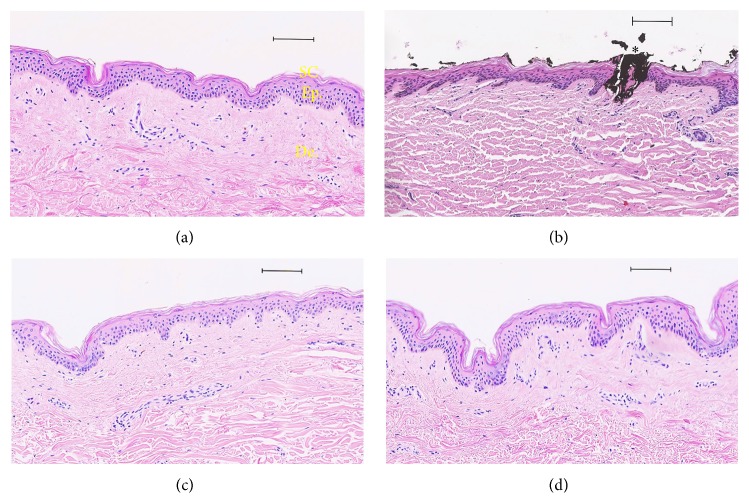
Histological analysis by optical microscopy (haematoxylin and eosin stain), comparison between a healthy human skin (a) and microneedles application with black ink (b), shaving human skin with a razor (c), and rotating brush and a gel (d). Scale bar: 100 *μ*m; SC: stratum corneum, Ep.: epidermis, and De.: dermis.

A statistically significant difference was determined (Table 3) between intact skin and punctured, brushed, shaved, and 30-stripped skins (KW was equal to 256.59, *P* value = 0.00). However, no difference could be detected between the skin samples when compared to differing subgroups of mechanical damage (Figure 5) following TEWL analysis. Histological findings of skin samples are shown in Figures 6 and 7. We have demonstrated that the lesions and functional changes induced by the 30-stripped skin model of barrier disruption are similar to those observed with devices examined.

Compared to intact skin, porcine biopsy holes (microlancing) are observed on the skin surface by SEM (Figure 6(a)). They are explained by a fracture from the stratum corneum into the dermis after the microneedles application (Figure 6(b), denoted by ^∗^). Moreover, the needles were soaked in black ink in order to avoid an artifact observation due to sample preparation. The same observation was made on human skin (Figure 7(b), denoted by ^∗^). Microneedles were used initially in the biopharmaceutical field for transdermal drug delivery in order to overcome the skin barrier by formation of mechanically produced conduits through the stratum corneum by the use of small needles [50–54].

Traditional methods of removing unwanted hair include shaving [55]; this method has a temporary impact on skin barrier function. This process removes the hair shaft very close to the surface of the skin as observed in Figure 6(c) (porcine biopsy). In contrast, no collateral damage to the softer skin surface was noticed for porcine and human skins (resp., in Figures 6(d) and 7(c)).

Microscopic examination of porcine skin after a rotative brush treatment depicts a very slight disturbance of epidermal tissue (Figures 6(e) and 6(f)). The same observation was made on human skin with rotating brush (Figure 7(d)). Consumers often combined the rotating brush with a cleansing gel as stimulated by our investigations. This technique is well known by dermatologists in the treatment of skin surface troubles like acne, scars, and other skin blemishes. It involves direct removal or disruption of the upper layer of the skin to enhance the penetration of topically applied xenobiotic [56]. From our observations, daily application of a cleansing gel with a device could be deleterious and could enhance percutaneous penetration of other chemicals applied onto the skin. In conclusion, based on data presented here, 30 tape-strips are necessary to obtain a model of realistic damage.

Numerous studies of TEWL or electrical resistance have compared healthy and damaged skin, but in each report a maximum of 10 or 20 tape-strippings were implemented to reenact disturbed skin. In contrast to our study, these tape-stripping models were not compared with other mechanical damage [48, 57, 58]. Although we observed a link between increases of TEWL absorption with numerous damage models, our results need to be further investigated in a quantitative fashion to appreciate the potential real life impact.

In our study, nonsignificant differences between mechanical damage and 30 tape-strips were demonstrated with reproducible data. This last procedure appeared to be a more realistic model in order to mimic human skin with impaired SC due to various mechanical reasons. We recommend a standardized method with 30 adhesive discs pressed onto the surface during 3 seconds using a 225 g/cm^2^ applicator to evaluate skin absorption for risk assessment.

With such a great enhancement of the TEWL found for all skin injuries, one can reasonably expect that skin absorption of chemicals would be similar following injury. A solution of 1% chlorogenic acid, a compound used as reference for skin absorption [59], was deposited on intact skin and two different types of damaged skins: 30 tape-strips and microneedles treatment. Absorption was qualitatively analyzed 24 hours after using fluorescent microscopy.

**Figure 8:**
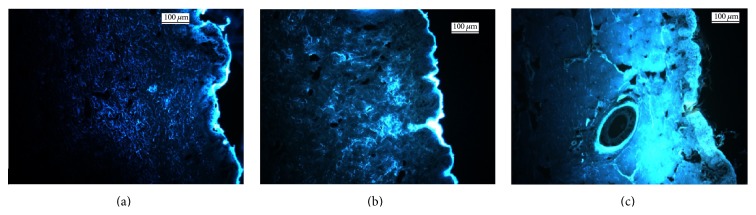
Fluorescence microscopy analysis: evaluation of penetration by chlorogenic acid (1% in solution), on healthy and damaged porcine skin. (a) Healthy porcine skin, (b) 30-time tape-stripped porcine skin, and (c) microneedles application. Scale bar: 100 *μ*m.

A bright high intensity fluorescence was clearly visible in upper layers of the intact skin, while a more diffuse signal was present at deeper skin layers (Figure 8). On the opposite, a wide area of fluorescence was observed deeper in the skin (Figures 8(b) and 8(c)) for both stripping method and microneedles. But, with the use of sharp microneedles, the diffusion of the fluorescent molecule through the conduits over time seemed to deeply penetrate the epidermis and the dermis. These results also suggested that a wide amount of chemicals could be absorbed, but more absorption studies are necessary to confirm our results.

## 4. Conclusion

Our objective was to determine a realistic and practical* in vitro* model of barrier impairment using a stepwise approach of sequential tape-stripping of pig and human skins in comparison to much mechanical damage currently encountered in the cosmetic field. TEWL was used to compare skin barrier function in human or pig skins. A dramatic increase of the TEWL value was observed with human skin with stretch marks compared to intact human skin. The experimental work presented herein has shown that the removal of stratum corneum by 30 tape-strips is the most relevant procedure in order to make a standardized model of injured skin* in vitro*. Skin exposed to microneedles, a razor, or a rotating brush was strongly disturbed and all the features of the damage were comparable to the 30 tape-strippings procedure in TEWL analysis, but we observe different kinds of skin barrier disruption. Results obtained in this work support the need for new absorption studies on damaged skin. Further perspectives are needed to answer further questions created in this study: how deep is the skin penetration for different compounds and what lies between healthy and damaged skins in this regard. This leads to the opening of investigations for the future, which questions the safety of advances in the development of topical formulations and cosmetic gadgetry in years to come.

## Figures and Tables

**Table 1:** TEWL data (number of experimentations with mean and median) obtained from healthy human (IntactHSkin) and porcine (IntactPigS.) skin biopsies and human stretch marks biopsies (StretchM.HSkin).

Type of skins	*n*	Average rank	Median (TEWL) g/m²/h	Mean (TEWL) g/m²/h
Intact pig skins	154	166.68	6.60	6.70 ± 0.23
Intact human skins	106	77.92	3.95	4.23 ± 0.28
Stretch marks skins	8	264.5	13.55	13.6 ± 0.58

**Table 2:** TEWL data (number of experimentations with mean and median) of stripped skin for 30 times.

Type of skins	*n*	Average rank	Median (TEWL) g/m²/h	Mean (TEWL) g/m²/h
Pig skins protocol 1—30 times	4	26.5	26.05	27.20 ± 2.77
Pig skins protocol 2—30 times	23	20.91	24.9	25.30 ± 1.15
Human skins protocol 1—30 times	10	23.75	27.3	26.03 ± 1.75
Human skins protocol 2—30 times	7	23.64	24.9	26.80 ± 2.09

**Table 3:** TEWL data (number of experimentations with mean and median) obtained with healthy human (IntactHSkin) and porcine (IntactPigS.) skins and human stretch marks biopsies (StretchM.HSkin).

Type of skins	*n*	Average rank	Median (TEWL) g/m²/h	Mean (TEWL) g/m²/h
Intact pig skin	154	166.68	6.60	6.70 ± 0.23
30-strip pig skin (pooled protocols 1-2)	27	302.01	25.70	25.58 ± 0.56
Brush gel pig skin	9	290.00	23.60	23.53 ± 0.87
Razor pig skin	6	340.50	35.35	33.96 ± 1.18
Microneedle pig skin	14	300.35	24.40	25.45 ± 0.77
Intact human skin	106	77.92	3.95	4.23 ± 0.28
30-strip human skin (pooled protocols 1-2)	17	306.29	26.90	26.34 ± 0.70
Brush gel human skin	5	319.10	29.50	28.78 ± 1.30
Razor human skin	8	328.68	30.08	30.82 ± 1.02
Microneedle human skin	79	312.83	30.00	27.67 ± 0.97

## References

[1] Marzulli F. N. (1962). Barriers to skin penetration. *The Journal of Investigative Dermatology*.

[2] Vieille-Petit A., Blickenstaff N., Coman G., Maibach H. (2015). Metrics and clinical relevance of percutaneous penetration and lateral spreading. *Skin Pharmacology and Physiology*.

[3] Smith W. (1999). Stratum corneum barrier integrity controls skin homeostasis. *International Journal of Cosmetic Science*.

[4] Scheuplein R. J. (2013). A personal view of skin permeation (1960–2013). *Skin Pharmacology and Physiology*.

[5] Norlén L. (2013). Current understanding of skin barrier morphology. *Skin Pharmacology and Physiology*.

[6] Hadgraft J. (1999). Passive enhancement strategies in topical and transdermal drug delivery. *International Journal of Pharmaceutics*.

[7] Singh S., Singh J. (1993). Transdermal drug delivery by passive diffusion and iontophoresis: a review. *Medicinal Research Reviews*.

[8] Junginger H. E. (1992). Formulation aspects on dermatological preparations and transdermal drug delivery systems. *Acta Pharmaceutica Nordica*.

[9] Caddeo C., Chessa M., Vassallo A. (2013). Extraction, purification and nanoformulation of natural phycocyanin (from *Klamath algae*) for dermal and deeper soft tissue delivery. *Journal of Biomedical Nanotechnology*.

[10] Ghanbarzadeh S., Arami S. (2013). Enhanced transdermal delivery of diclofenac sodium via conventional liposomes, ethosomes, and transfersomes. *BioMed Research International*.

[11] Alexander A., Dwivedi S., Giri T. K., Saraf S., Tripathi D. K. (2012). Approaches for breaking the barriers of drug permeation through transdermal drug delivery. *Journal of Controlled Release*.

[12] Park J.-H., Choi S.-O., Seo S., Choy Y. B., Prausnitz M. R. (2010). A microneedle roller for transdermal drug delivery. *European Journal of Pharmaceutics and Biopharmaceutics*.

[13] Andre O., Paye M., Maibach H. I. (2009). *Handbook of Cosmetic Science and Technology*.

[14] (2004). *OECD Guideline for the Testing of Chemicals, Test No. 428: Skin Absorption: In Vitro Method*.

[15] Jacobi U., Kaiser M., Toll R. (2007). Porcine ear skin: an *in vitro* model for human skin. *Skin Research and Technology*.

[16] Darvin M. E., Richter H., Zhu Y. J. (2014). Comparison of *in vivo* and *ex vivo* laser scanning microscopy and multiphoton tomography application for human and porcine skin imaging. *Quantum Electronics*.

[17] Roetman E. L., Barr R. E. (1976). The mechanical basis for Fick's law and its generalizations. *Advances in Experimental Medicine and Biology*.

[18] Couto A., Fernandes R., Cordeiro M. N. S., Reis S. S., Ribeiro R. T., Pessoa A. M. (2014). Dermic diffusion and stratum corneum: a state of the art review of mathematical models. *Journal of Controlled Release*.

[19] Fritsch P. O., Gschnait F., Kaaserer G. (1979). PUVA suppresses the proliferative stimulus produced by stripping on hairless mice. *Journal of Investigative Dermatology*.

[20] Breternitz M., Flach M., Prässler J., Elsner P., Fluhr J. W. (2007). Acute barrier disruption by adhesive tapes is influenced by pressure, time and anatomical location: integrity and cohesion assessed by sequential tape stripping; a randomized, controlled study. *British Journal of Dermatology*.

[21] Berrutti L. E., Singer A. J., McClain S. A. (2000). Histopathologic effects of cutaneous tape stripping in pigs. *Academic Emergency Medicine*.

[22] Dickel H., Goulioumis A., Gambichler T. (2010). Standardized tape stripping: a practical and reproducible protocol to uniformly reduce the stratum corneum. *Skin Pharmacology and Physiology*.

[23] Peppelman M., van den Eijnde W. A., Jaspers E. J., Gerritsen M. P., van Erp P. E. (2015). Combining tape stripping and non-invasive reflectance confocal microscopy: an *in vivo* model to study skin damage. *Skin Research and Technology*.

[24] Yamamura T., Tezuka T. (1988). A new technique for measuring trans-epidermal water loss (TEWL). *Nihon Hifuka Gakkai Zasshi*.

[25] Pinnagoda J., Tupker R. A., Agner T., Serup J. (1990). Guidelines for transepidermal water loss (TEWL) measurement. A report from the Standardization Group of the European Society of Contact Dermatitis. *Contact Dermatitis*.

[26] Serup J., Guilhou J. J. (1995). TEWL measurement standardization. *Acta Dermato-Venereologica*.

[27] Hattingh J. (1972). A comparative study of transepidermal water loss through the skin of various animals. *Comparative Biochemistry and Physiology A: Molecular & Integrative Physiology*.

[28] Guth K., Schäfer-Korting M., Fabian E., Landsiedel R., van Ravenzwaay B. (2015). Suitability of skin integrity tests for dermal absorption studies *in vitro*. *Toxicology in Vitro*.

[29] Lodén M. (2003). Role of topical emollients and moisturizers in the treatment of dry skin barrier disorders. *American Journal of Clinical Dermatology*.

[30] Werner Y. L. V. A., Lindberg M. (1985). Transepidermal water loss in dry and clinically normal skin in patients with atopic dermatitis. *Acta Dermato-Venereologica*.

[31] Grice K., Sattar H., Baker H., Sharratt M. (1975). The relationship of transepidermal water loss to skin temperature in psoriasis and eczema. *Journal of Investigative Dermatology*.

[32] Blattner C. M., Coman G., Blickenstaff N. R., Maibach H. I. (2014). Percutaneous absorption of water in skin: a review. *Reviews on Environmental Health*.

[33] Kochhar J. S., Quek T. C., Soon W. J., Choi J., Zou S., Kang L. (2013). Effect of microneedle geometry and supporting substrate on microneedle array penetration into skin. *Journal of Pharmaceutical Sciences*.

[34] Akomeah F. K., Martin G. P., Muddle A. G., Brown M. B. (2008). Effect of abrasion induced by a rotating brush on the skin permeation of solutes with varying physicochemical properties. *European Journal of Pharmaceutics and Biopharmaceutics*.

[35] Kircik L. H. (2014). A study to assess the occlusivity and moisturization potential of three topical corticosteroid products using the skin trauma after razor shaving (STARS) bioassay. *Journal of Drugs in Dermatology*.

[36] Neu R. (1957). A new reagent for differentiating and determining flavones on paper chromatograms. *Naturwissenschaften*.

[37] Manugistics (2014). *Statgraphics Plus for Windows*.

[38] Mitts T. F., Jimenez F., Hinek A. (2005). Skin biopsy analysis reveals predisposition to stretch mark formation. *Aesthetic Surgery Journal*.

[39] Sekkat N., Kalia Y. N., Guy R. H. (2002). Biophysical study of porcine ear skin in vitro and its comparison to human skin in vivo. *Journal of Pharmaceutical Sciences*.

[40] Debeer S., Le Luduec J. B., Kaiserlian D. (2013). Comparative histology and immunohistochemistry of porcine versus human skin. *European Journal of Dermatology*.

[41] Netzlaff F., Kostka K.-H., Lehr C.-M., Schaefer U. F. (2006). TEWL measurements as a routine method for evaluating the integrity of epidermis sheets in static Franz type diffusion cells *in vitro*. Limitations shown by transport data testing. *European Journal of Pharmaceutics and Biopharmaceutics*.

[42] Rubio L., Alonso C., López O. (2011). Barrier function of intact and impaired skin: percutaneous penetration of caffeine and salicylic acid. *International Journal of Dermatology*.

[43] Trebilcock K. L., Heylings J. R., Wilks M. F. (1994). In vitro tape stripping as a model for in vivo skin stripping. *Toxicology in Vitro*.

[44] Gao Y., Wang X., Chen S., Li S., Liu X. (2013). Acute skin barrier disruption with repeated tape stripping: an in vivo model for damage skin barrier. *Skin Research and Technology*.

[45] Escobar-Chávez J. J., Merino-Sanjuán V., López-Cervantes M. (2008). The tape-stripping technique as a method for drug quantification in skin. *Journal of Pharmacy and Pharmaceutical Sciences*.

[46] Fernandez C., Nielloud F., Fortuné R., Vian L., Marti-Mestres G. (2002). Benzophenone-3: rapid prediction and evaluation using non-invasive methods of in vivo human penetration. *Journal of Pharmaceutical and Biomedical Analysis*.

[47] Bettoni C. C., Felippi C. C., De Andrade C. (2012). Isotretinoin-loaded nanocapsules: stability and cutaneous penetration by tape stripping in human and pig skin. *Journal of Biomedical Nanotechnology*.

[48] Davies D. J., Heylings J. R., McCarthy T. J., Correa C. M. (2015). Development of an *in vitro* model for studying the penetration of chemicals through compromised skin. *Toxicology in Vitro*.

[50] Tuan-Mahmood T.-M., McCrudden M. T. C., Torrisi B. M. (2013). Microneedles for intradermal and transdermal drug delivery. *European Journal of Pharmaceutical Sciences*.

[51] Henry S., McAllister D. V., Allen M. G., Prausnitz M. R. (1998). Microfabricated microneedles: a novel approach to transdermal drug delivery. *Journal of Pharmaceutical Sciences*.

[52] Prausnitz M. R. (2004). Microneedles for transdermal drug delivery. *Advanced Drug Delivery Reviews*.

[53] Gerstel M. S., Place V. A. Drug delivery device.

[54] Verbaan F. J., Bal S. M., van den Berg D. J. (2007). Assembled microneedle arrays enhance the transport of compounds varying over a large range of molecular weight across human dermatomed skin. *Journal of Controlled Release*.

[55] Cowley K., Vanoosthuyze K. (2012). Insights into shaving and its impact on skin. *British Journal of Dermatology*.

[56] Brown M. B., Traynor M. J., Martin G. P., Akomeah F. K. (2008). Transdermal drug delivery systems: skin perturbation devices. *Methods in Molecular Biology*.

[57] Zhai H., Dika E., Goldovsky M., Maibach H. I. (2007). Tape-stripping method in man: comparison of evaporimetric methods. *Skin Research and Technology*.

[58] Klang V., Schwarz J. C., Lenobel B. (2012). *In vitro* vs. *in vivo* tape stripping: validation of the porcine ear model and penetration assessment of novel sucrose stearate emulsions. *European Journal of Pharmaceutics and Biopharmaceutics*.

[59] Marti-Mestres G., Mestres J. P., Bres J., Martin S., Ramos J., Vian L. (2007). The ‘*in vitro*’ percutaneous penetration of three antioxidant compounds. *International Journal of Pharmaceutics*.

